# Natural History Of an Untreated Type 1 Endoleak: A Case Report

**DOI:** 10.7759/cureus.1507

**Published:** 2017-07-24

**Authors:** Alice Shen, Gabriel O Ologun, Hilary Keller, Lawrence Sampson

**Affiliations:** 1 General Surgery, Guthrie Clinic/Robert Packer Hospital; 2 Vascular Surgery, Guthrie Clinic/Robert Packer Hospital

**Keywords:** endovascular aneurysm repair (evar), endoleak, abdominal aortic aneurysm (aaa)

## Abstract

We present a case of giant abdominal aortic aneurysm greater than 17 cm complicated by an endoleak, demonstrating the natural history of an untreated Type 1 endoleak.

## Introduction

Abdominal aortic aneurysms are the most common type of aortic aneurysm seen in 3% - 9% of patients ages greater than 50 years in the Western world. Ruptured abdominal aortic aneurysms are the 10th leading cause of death in men above 55 years old [1]. The advancement of endovascular aneurysm repair has lowered the perioperative mortality compared to open repair. We present a case of a giant abdominal aortic aneurysm after endovascular repair. Informed consent was obtained for this case report, images, and publication.

## Case presentation

The patient is a 67-year-old male with a history of hypertension, chronic obstructive pulmonary disease, and a known infrarenal abdominal aortic aneurysm (AAA) who presented to the emergency department with abdominal pain of about six to eight hours duration. He was on antihypertensive, statin, and antiplatelet medications. He developed sudden sharp pains in his mid to lower abdomen. He had moderate pain. He denied nausea, vomiting, diarrhea, melena, or hematochezia.

Of note, the patient had an open AAA repair with a graft for rupture with subsequent AAA growth secondary to endotension, resulting in an endovascular revision and stent placement at an outside facility. He had been followed in our institution's vascular department for over a year for surveillance per request from his primary physician due to the gradual growth in the size of his AAA. Treatment options were discussed, including open versus endovascular approaches, plus a second opinion was obtained from another vascular surgeon in our facility. The patient declined any surgical or endovascular intervention, accepting his risk of mortality from sudden rupture.

This patient’s vital signs were within normal ranges, except for a sinus tachycardia of 104 and systolic blood pressure ranging from 90 - 110. On examination, the patient was uncomfortable. He had biphasic Doppler signals in his distal lower extremities bilaterally. The abdomen was softly distended and mildly tender with an appreciable pulsatile mass.

**Figure 1:**
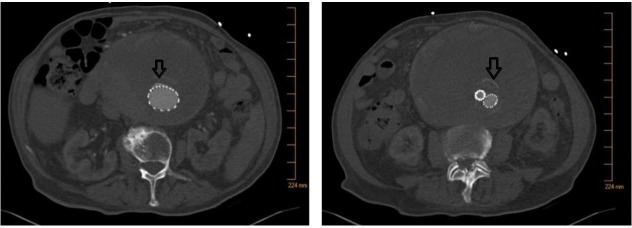
Axial computed tomographic angiography of an abdominal aortic aneurysm greater than 17 cm with arrow showing the endoleak

A computed tomographic angiogram (CT angio) demonstrated an AAA with evidence of interval increase in size from 16.3 cm to 17.3 cm within the past six months with evidence of a Type 1 endoleak identified near the central portion of the aortic graft (Figure 1). At this point, the vascular surgery team was consulted to evaluate the patient.

Computed tomographic angiography (CT angio) results were discussed with the patient and his family. The patient again declined against any surgical intervention. He opted for palliative/comfort care measures. Shortly thereafter, the patient became hypotensive, unconscious, and pulseless, and he was pronounced dead at that time. The family declined an autopsy.

## Discussion

This is an interesting case presentation of the natural history of an untreated Type 1 endoleak noted on CT angio. AAA is the most common type of aortic aneurysm and is seen in 3% to 9% of patients over the age of 50 years in the Western world [1]. Since the introduction and rapid advancement of endovascular aneurysm repair (EVAR) in the 1990s, multiple prospective randomized trials have shown that the endovascular technique lowers the 30-day perioperative mortality in contrast to open repair (1.7% versus 4.7%, respectively); the all-cause mortality was not different [2-5]. However, EVAR  has a higher long-term complication rate than open AAA repair, and endoleaks are unique complications. Endoleaks are defined as persistent blood flow in an aneurysm outside the prosthesis, causing enlargement of the aneurysm sac. They have been classified into five types according to etiology. They are summarized as the following:

Type I: Leakage from the proximal or distal anchoring points from an ineffective seal Type II: Leakage from the retrograde flow through the lateral branches (e.g., lumbar artery or inferior mesenteric artery) not covered by the graft. This is the most common type. Type III: Leakage from the overlap zones of the individual stent prosthesis components Type IV: Leakage through the stent material, now rare as a result of improved stent prosthesis Type V: “Endotension” seen on follow-up imaging as an increase in aneurysm size without contrast extravasation outside of the stent prosthesis itself. This is thought to be caused by increased pressure.

Patients who have had EVAR require lifelong surveillance because endoleaks can lead to aneurysm expansion and rupture. Each endoleak type has its own algorithm of intervention directed at treating the underlying etiology. Type I and III endoleaks represent direct communication with the systemic blood flow and require immediate repair. They can be approached endovascularly or via conversion to an open procedure. Type II endoleaks can spontaneously thrombose and immediate intervention is not needed in an asymptomatic patient. Type IV endoleaks require stent replacement, but this has been rare due to the improved prosthetic material. Type V endoleaks often require conversion to open repair, but continued nonoperative management is also acceptable [6].

## Conclusions

This case presents a patient with a giant AAA secondary to a Type I endoleak after a prior open repair who declined any surgical intervention and ultimately succumbed to his disease. Despite the large aneurysm size and continued expansion, this patient was asymptomatic and survived more than seven years after the endovascular revision of his previous open AAA repair. Moreover, this patient survived 17 years after the initial ruptured native AAA that required open repair. The risk of revisional surgery versus the risk of rupture with watchful waiting in elderly patients remains an ongoing debate.
